# Ancel Keys: a tribute

**DOI:** 10.1186/1743-7075-2-4

**Published:** 2005-02-14

**Authors:** Theodore B VanItallie

**Affiliations:** 1 Department of Medicine, St Luke's/Roosevelt Hospital Center, Columbia University College of Physicians and Surgeons, New York, NY, USA

## Abstract

Ancel Keys, Ph.D., who died in November, 2004, at the age of 100, was among the first scientists to recognize that human atherosclerosis is not an inevitable consequence of aging, and that a high-fat diet can be a major risk factor for coronary heart disease. During World War II, he and a group of talented co-workers at the University of Minnesota conducted a large-scale study of experimentally-induced human starvation. The data generated by this study – which was immediately recognized to be a classic – continue to be of inestimable value to nutrition scientists. In his later years, Keys spent more time at his home in Naples, Italy, where he had the opportunity to continue his personal study of the beneficial effects on health and longevity of a Mediterranean diet.

Ancel Keys, who died in November, 2004, was an excellent testimonial to the health-promoting effects of his beloved Mediterranean diet. He lived to be 100 and, as the *New York Times *obituary put it, "remained intellectually active through his 97^th ^year." His latter years were spent mostly at his home in Naples, ItaIy. I never had the privilege of knowing him well, but encountered him occasionally at scientific meetings where we were both speakers. He was friendly but, I thought, reserved. What struck me about Ancel was his remarkable absence from the counsels of the nutrition establishment. Despite his acknowledged expertise and importance in the field, he was not a member of AMA's Council on Foods & Nutrition (at least not during the many years I served on that organization). I never saw him at any of the NIH advisory committees on which I served. He did not play a role in the deliberations of the Food and Nutrition Board of the National Research Council. He was not involved in the American Society for Clinical Nutrition during its heyday. Why was this? Perhaps the fact that he was a physiologist (later an epidemiologist) and not a physician played some role. Also, I think he preferred to go his own way, and – to some extent – he remained aloof from "academic nutrition." Yet he was willing to lecture to many audiences and was not considered to be a scientific eccentric; to the contrary, his epidemiological work was frequently cited and praised, and his monumental study of experimentally-induced semistarvation in human subjects [1] was immediately recognized to be a classic.

Keys and his capable associates conducted careful physiological and psychological studies of 32 initially healthy conscientious objectors (to World War II) through 6 months of experimentally induced semistarvation, followed by a year or more of rehabilitation. These studies generated a cornucopia of data – data that are all the more valuable now because such an experiment would not have a chance of being approved by today's Institutional Review Boards. Protein-calorie malnutrition (PCM) – in effect, famine – remains endemic in many parts of the world; moreover, PCM is the most common nutritional problem encountered in U.S. hospitals and nursing homes. The studies carried out by Keys and his co-workers make it possible for us to distinguish the effects of semistarvation on the body's strength, composition, physiological status, and mood from the confounding effects of such underlying diseases as cancer, intestinal malabsorption, renal insufficiency, emphysema, etc. – illnesses that often give rise to conditioned PCM. The Minnesota group showed clearly that semistarvation can be independently responsible for an array of psychological problems such as anxiety, depression, and hypochondria. From their studies, it is possible to demonstrate a clear relationship between a decline in fat-free mass and PCM-associated morbidity.

Keys's major scientific achievements are enumerated in some detail by Jane E. Brody in her *New York Times *obituary, dated November 23, 2004. For those of us who worked for so many years to call attention to the relationship of serum total cholesterol to risk of coronary heart disease (CHD), and to the cholesterol-raising effects of certain saturated fats, Keys will always be one of the major prophets who provided the early evidence that atherosclerosis is not an inevitable concomitant of aging, and that a diet high in saturated fat content can be a major risk factor for CHD. The practical outcome of the work in this field – to which Ancel contributed so much – is the extraordinary decrease in mortality from coronary heart disease that has occurred during the past half-century. Cancer has finally replaced heart disease as America's number one killer. Ancel had his well-deserved reward – a long, productive life unencumbered by an excess of committee meetings, and the opportunity to contemplate the Tyrrhenian sea while enjoying the benefits of a Mediterranean diet.
